# Development and Evaluation of Interactive Flipped e-Learning (iFEEL) for Pharmacy Students during the COVID-19 Pandemic

**DOI:** 10.3390/ijerph19073902

**Published:** 2022-03-25

**Authors:** Ahmad A. Shahba, Zaid Alashban, Ibrahim Sales, Abdelrahman Y. Sherif, Osman Yusuf

**Affiliations:** 1 Department of Pharmaceutics, College of Pharmacy, King Saud University, Riyadh 11451, Saudi Arabia; zalashban@ksu.edu.sa (Z.A.); ashreef@ksu.edu.sa (A.Y.S.); osmohamed@ksu.edu.sa (O.Y.); 2 Department of Clinical Pharmacy, College of Pharmacy, King Saud University, Riyadh 11451, Saudi Arabia; isales@ksu.edu.sa; 3 Kayyali Chair for Pharmaceutical Industries, College of Pharmacy, King Saud University, Riyadh 11451, Saudi Arabia

**Keywords:** flipped learning, interactive e-lectures, distance learning, learning during COVID-19 pandemic

## Abstract

Background: Distance learning has come to the forefront of educational delivery throughout the world due to the COVID-19 pandemic. Presently, there is a paucity of studies that have utilized interactive e-lectures as a model for remote flipped learning. Objectives: To compare educational outcomes for the remote interactive flipped e-learning (iFEEL) activity versus paper-based in-class group learning (PICkLE). Methods: During the spring 2021 semester, tutorials in pharmaceutical quality control and good manufacturing practice were remotely delivered to students by two different approaches: PICkLE and iFEEL. In the latter activity, interactive e-lectures were software-designed and included several audiovisual enhanced illustrations to encourage students to interact with the lecture material prior to attending the virtual class. The class time was reserved for in-class quizzes and discussion. Mean exam scores were compared and voluntary questionnaires were distributed among the participating students as well as healthcare faculty members in 29 Saudi universities. Data from the remotely-delivered course was compared with data from previous course offerings (2018–2020) that used the live PICkLE method. Results: The mean score of post-lecture tests significantly (*p* < 0.05) increased compared to pre-lecture tests in remote PICkLE and iFEEL, respectively. iFEEL activity showed higher mean post-tests score (95.2%) compared to live PICkLE (90.2%, *p* = 0.08) and remote PICkLE (93.5%, *p* = 0.658). Mean comprehensive exam scores increased from 83.8% for remote PICkLE to 89.2% for iFEEL (*p* = 0.449). On average, 92% of students and 85% of faculty members reported positive feedback on the five quality attributes of the e-lecture. Over 75% of students preferred the iFEEL over PICkLE activity for future course offerings and 84% of faculty members recommend the integration of interactive e-lectures in their future courses. Conclusion: iFEEL represents a novel model of remote flipped learning and shows promising potential to be incorporated into live blended-learning classroom activities.

## 1. Introduction

The COVID-19 pandemic led to a sudden suspension of face-to-face teaching activities across the world [1]. Immense school closures in more than 200 countries have displaced approximately 1.6 billion learners, equivalent to more than 94% of the student population all over the globe [2,3]. The pandemic forced academic institutions to immediately shift to virtual education or online learning as an emergent solution [1,2,4]. In. In response to such an urgent transition, faculty and educators adopted several strategies to facilitate online learning such as providing educational resources for self-study [2], pre-recorded lecture videos [1], video conferencing, interactive online simulation programs [2], flipped and flipped-jigsaw learning [5,6,7], online problem-based learning [8,9], and synchronous and asynchronous online discussions [2]. Alternative strategies for courses with clinical/practical aspects involved posting clinical case scenarios and/or uploading ready-made videos covering the required topic [2].

In particular, the implementation of the flipped classroom shifts the educator’s role towards that of a facilitator who promotes problem solving, rather than delivering didactic knowledge. However, the online format of flipped classrooms should be carefully implemented to avoid negative learning outcomes. Cho and Kim reported inferior self-directed learning readiness and professor–student interaction when comparing online to face-to-face flipped learning [10]; therefore, these parameters should be carefully considered when implementing online flipped classrooms. On the other hand, Smith and Boscak utilized the flipped classroom mode of instruction in restructuring a course to an online format [5]. They reported successful course delivery with good learning outcomes, highlighting the merits of self-directed learning and flipped-classroom techniques. Duszenko et al. implemented a flipped classroom model in a digital training course [11]. In this study, most students favored the flipped classroom format and 52% acknowledged that the flipped classroom was very helpful. The students preferred the simplistic explanations of their peers as opposed to instructor-led learning and perceived that this method enabled them to identify incomplete or misunderstood information [11]. Ketterer et al. reported improved learning outcomes after implementing an online case-based curriculum in a flipped classroom design [7]. Although flipped learning requires considerable preparation on the instructor’s part, it results in a high-yield educational impact with less instructional time [12].

In fact, several efforts have been investigated to determine the best means to actively engage healthcare learners remotely [13,14]. Pilkington and Hanif preferred short pre-recorded lecture videos rather than live streaming of lectures [1]. After reviewing the lectures, students were then allowed to attend live tutorial sessions if they had any questions that they wanted to ask the lecturer directly. Suppan et al. described the development of a storyline e-learning module for healthcare professionals to provide education about the proper use of personal protective equipment during the pandemic [15]. However, these online education platforms have completely different requirements, structures, and contexts compared to traditional face-to-face education. Most importantly, success in these online learning activities requires a high degree of student self-direction, motivation, and interaction [16].

During the COVID-19 pandemic, some preliminary studies have reported that the sudden transition to online learning has been well accepted [17,18,19]. Some students reported positive-rated aspects of online learning, such as flexibility, affordability [20], higher motivation, less time effort, and that online learning was even easier compared to “face-to-face” courses [21].

However, many other studies [22,23,24] have emphasized the challenges that many students face such as low concentration in online learning [22], lack of engagement in online classes [25], lack of preparedness [20,21], procrastination and self-regulation [20,26,27], poor time management [28], feelings of isolation, low motivation, high stress and anxiety [23] digital divide, and technical difficulties [20,29,30]. On the other hand, teachers have reported the transition to online learning being time constraining and demanding more preparation, better classroom management [24,31] as well as the difficulty to work from home [21], and limited supervision on online assessments [29]. Students and faculty with low digital competence found it difficult to make optimal utilization of online learning [29].

Another challenge that faced both students and educators, was the unprecedented load on the Internet network [30]. Live classrooms placed a large workload on teaching servers with significant peaks on scheduled lecture times; while on-demand classrooms and teaching materials access were spread over the day. Some students and/or educators suffered eventual internet impairment which is mainly critical for synchronous online communication. On the other hand, asynchronous learning environments such as on-demand classrooms and pre-recorded videos appear to be less susceptible to internet impairments but lack the real-time interactions between educators and learners [20].

Therefore, innovative solutions were needed to circumvent such challenges and enhance online learning quality during the COVID-19 pandemic. Dhawan emphasizes that online courses should be dynamic, interactive, student-centered, and interesting [20]. Adedoyin and Soykan highlighted the importance of developing equitable and unbiased online assessment systems to minimize risks of cheating and plagiarism [29]. Khtere and Yousef concluded that the instructor plays a principal role in achieving professional online learning outcomes by acting as a guide and motivator for students, an instructional designer who utilizes technology to enhance the quality of learning, and as a sincere advisor who provides effective feedback, follow-up, and necessary recommendations to students [3].

According to the previously discussed limitations of online learning, there is an urgent need to develop e-learning techniques that are interactive, user-friendly, foster student engagement in the learning process, avoid technical issues, and that are compatible with various digital devices.

As a response to the global lockdown situation, the Center for Excellence in Learning and Teaching at King Saud University (KSU) announced the launch of the fifth cycle of the Excellence in Teaching and Learning Grants Program. This cycle targeted the most current topics in distance learning involving e-learning activities, online/remote assessments, and reusable electronic learning resources.

Therefore, we introduced an interactive e-learning activity for pharmacy students to enable them to continue their education remotely with a high level of interaction and enhanced learning outcomes. To the best of our knowledge, interactive e-lecture flipped learning (iFEEL) is an active learning model that was applied for the first time in our college. It was implemented to evaluate the potential of this method to keep students focused during distance learning and encourage them to review the lecture information electronically prior to attending the virtual lecture [32]. The objective of this study was to introduce the iFEEL activity into the Pharmaceutical Quality Control and Good Manufacturing Practice (GMP) course and compare its educational outcomes against paper-based in-class group learning (PICkLE). Student scores, perceptions, and preferences for teaching modality were compared to determine the most effective strategy.

Important questions that can be addressed by the current study include: “how to motivate/engage students in online learning” and “how to ensure proper knowledge transfer”. Moreover, it is important to explore which novel teaching modalities should be permanently anchored in online learning. The overall goal of this study was to obtain a qualitative and quantitative assessment of the newly developed iFEEL activity by students and teachers in terms of effectiveness, quality assurance, and in comparison with the previously delivered live courses [11].

## 2. Methods

### 2.1. Study Design

This was a pilot study that utilized a mixed-methods design [33] and predominately fit the ADDIE (Analysis, Design, Development, Implementation, Evaluation) theoretical framework [34]. The study was conducted in the College of Pharmacy at KSU (Level 10, Bachelor’s degree (B. Pharm), male campus). At the time the study was conducted, the KSU College of Pharmacy was in the final stages of transitioning from offering both the B. Pharm and the Doctor of Pharmacy (PharmD) degrees to a single terminal degree—the PharmD. The Pharmaceutical Quality Control and GMP course (tutorials) was selected to pilot the iFEEL activity in the spring 2021 semester. In addition, data from the past five live offerings of this course (2018–2020) were retrospectively collected and compared with the data from the current remote delivery of the course. This course covers important pharmaceutical quality control tests with an overview of the various pharmacopoeias. By the completion of this course, the students should gain the basic knowledge and skills which they need to gain employment in the quality control of pharmaceutical products. Examples of topics covered by the course include friability, disintegration, dissolution tests, uniformity of dosage units, quality control of glass containers, and pyrogen and particulate matter tests.

### 2.2. Software and Applications

A variety of software and applications were utilized to facilitate student learning, comprehension, and online assessment. Blackboard^®^ is the standard learning management system provided by KSU. Storyline 360^®^, an e-learning authoring application designed by Articulate Inc., New York, USA, and Camtasia^®^ 2020 Education, a screen recording and video editing software, developed by TechSmith Corporation, Okemos, MI, USA, were utilized to design the e-lectures. Google Forms^®^ software was provided as part of the free, web-based Google Docs Editors suite offered by Google.

### 2.3. Procedure

**Figure 1:**
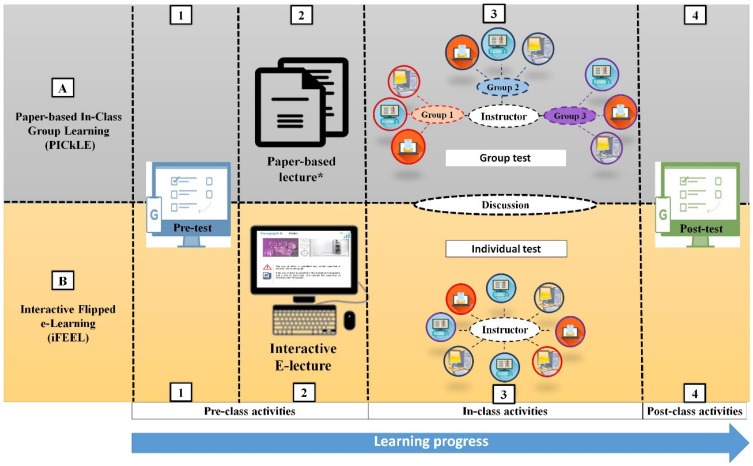
Graphical representation of the execution steps of (**A**) PICkLE and (**B**) iFEEL activities. * The students were informed that the lecture material was intended to be studied during the virtual classroom.

Due to COVID-19 pandemic precautions and according to the Saudi Arabian Ministry of Education’s directions, the course was delivered remotely. In the KSU-College of Pharmacy, cloud meetings software (such as Zoom meetings and Blackboard collaborate) was the standard method to deliver didactic lectures during the COVID-19 pandemic. Alternative methods also included posting recorded videos or PowerPoint presentations. These methods were primarily instructor focused and keeping students’ attention during the lecture could not be ensured or monitored. However, the current study utilized two self-learning interactive approaches namely: (A) paper-based (PICkLE) and (B) and interactive e-lecture (iFEEL) as shown in Figure 1.

In the PICkLE activity, a pre-class online test was distributed to students before each lecture to assess their baseline knowledge of the lecture information (Figure 1, Steps A1). The lecture material for PICkLE was provided in the form of printable PDF files distributed among the students. The students were informed that this material was intended to be studied during the virtual classroom (Step A2). During the class, students were instructed to read the lecture material and solve an open-book online exam in groups (Step A3). The instructor’s role involved answering the students’ inquiries on difficult/unclear lecture information, assessing the students’ answers immediately to evaluate their understanding of the lecture material, and emphasizing the common mistakes rather than delivering the lecture information in the traditional didactic method. After the classroom ended, the students took a post-class online test which was prepared in a method similar to the pre-test (A4).

In the iFEEL activity, the students took an online pre-test (step B1), and then the students were instructed to review the e-lecture and answer the questions embedded in the lecture prior to the class (Step B2). During the class, students were instructed to solve an open-book online exam, individually (Step B3). Similar to PICkLE, the instructor’s role involved resolving the students’ inquiries and emphasizing common mistakes. The students took the post-class test after the class ended (B4).

The course consisted of ten lectures (tutorials). The first five lectures were delivered using PICkLE (as a control) while the remaining five lectures were delivered using iFEEL. In addition, the lectures assigned for the iFEEL were closely related and were considered to be more suitable to be delivered by the audiovisual enhanced iFEEL method. The students were provided with the course lecture materials in printable PDF format at the beginning of the semester (i.e., the 1st lecture).

In previous course offerings before COVID-19, the PICkLE method had successfully been used with high levels of student acceptance. In the current study, students were divided into groups of three members to read lecture material and take the open-book online exam during each virtual lecture [35]. The open-book exam was designed using Google^®^ forms to give prompt feedback to the students regarding their scores [35]. Subsequently, the instructor was able to view the students’ results immediately to assess their understanding of the lecture material, emphasize the common mistakes, and summarize the important information.

The iFEEL is a novel application of flipped learning (FL) using software-designed interactive e-lectures that were made accessible to students at least 24 h before the virtual lecture. The e-lectures were designed by Storyline 360^®^ in the form of interactive slides that contained a written explanation of the lecture information, computerized audio narrations, animated photos/illustrations, and/or educational videos. In addition, the e-lectures were enriched with hyperlinks to provide extra supportive audio/visual explanations of difficult/important information. Most importantly, the lecture slides included numerous interactive questions distributed throughout the lecture. These interactive questions provided the students with prompt feedback/scoring to assess their comprehension and increase their interaction with the scientific material. If the student missed the correct answer, he was usually provided with (1–3) chances/trials until he answered the question correctly. In certain situations, hints were provided to help the student discover the correct answer if they missed it on the first attempt. Besides the standard multiple-choice and fill-in-the-blank questions, some interactive question types such as dragging and dropping, clicking on the correct spot in the picture, watching a video and discovering the mistake, arranging steps in correct order along with audio/visual-enhanced supporting tools were embedded to encourage critical thinking. The educational videos of the lectures were voiced over and edited by the CAMTASIA^®^ program, whenever needed. The e-lecture involved a course progress icon to encourage students to complete the lecture. At the end of the lecture, the students were provided their cumulative score, immediately.

The e-lectures were designed with a restriction feature to prevent the student from skipping any slide and/or question. The student was able to move to the next slide only if he spent the pre-determined time on the slide and/or interacted with the required slide items.

**Figure 2:**
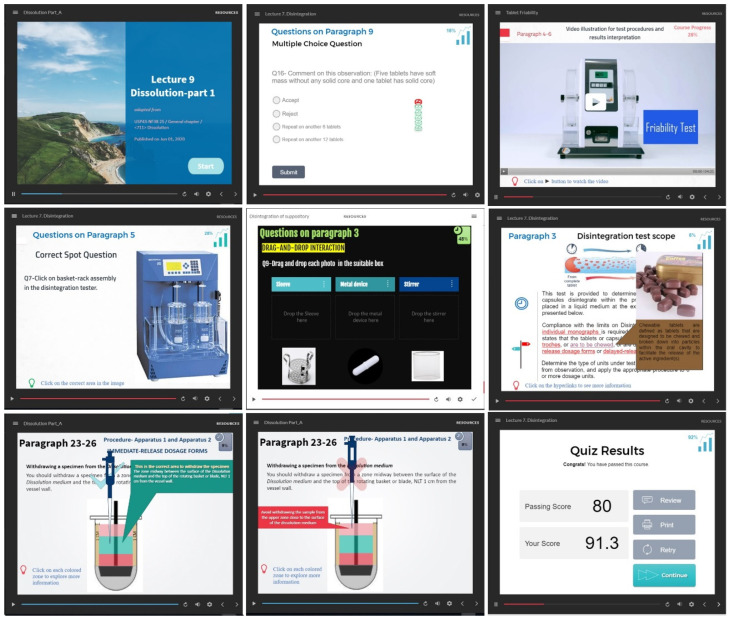
Captured screenshots of the e-lecture (iFEEL) slides.

The iFEEL activity was the first interactive e-lecture applied in this course. Therefore, a training video (in Arabic) was designed by the instructors and sent to the students prior to starting the iFEEL model to illustrate the e-lecture features and explain the proper way to interact with them. Captured screenshots of the e-lecture slides are presented in Figure 2. In addition, a model example of the e-lecture can be accessed through the following link: https://360.articulate.com/review/content/5f0c0097-d4ce-4393-a9e5-1793c3140d19/review (accessed on 17 March 2022).

In iFEEL, prior to attending the virtual lecture, the students had to complete the e-lecture and answer all the embedded questions with a minimum of 80% score. Each student was given unlimited attempts to obtain the required score. During the lecture, each student took an in-class open exam individually. Subsequently, the instructor discussed the results, emphasized the common mistakes, and summarized the important information. Student participation was determined by whether or not the student completed the e-lecture and got a minimum of 80% score in the embedded lecture questions, by the specified deadline.

The results of the PICkLE and iFEEL in-class quizzes were not subjected to score comparison due to the major differences between the testing methods (individual vs. group) in each technique; however, student comprehension was assessed by using other methods.

In the current course offering, each lecture, both PICkLE and iFEEL, involved a short pre-class test (pre-test) (Figure 1—Steps A1 and B1) and a post-class test (post-test) (Figure 1—Steps A4 and B4) to evaluate the effect of each learning activity on students’ comprehension of the lecture material. The pre- and post-tests were designed and made available to the students through Blackboard^®^. The post-test was predominately designed using the “question bank” feature to randomly select three different questions for each student. This step was undertaken to decrease the possibility of any increased scores due to memorization or screen capturing of the pre-test questions [36]. Only the post-test scores served as a reflection of the students’ overall performance and comprehension.

After completing the lectures for each learning method, the students took a paper-based open-book comprehensive exam on campus. Each of the two exams covered only the related lectures for the learning method.

The pre- and post-tests related to the lectures conducted with the PICkLE and iFEEL activities were grouped and analyzed separately. In addition, the results from students’ marks during the midterm exam (PICkLE activity), and the final exam (iFEEL activity) were compared to evaluate learning outcomes and student performance for each technique. In addition, the mean post-test scores of the past five live offerings of this course (2018–2020) were retrospectively collected and compared with remote PICkLE and iFEEL corresponding data. The exams results were statistically analyzed to determine whether there was a significant difference between these activities in terms of student scores.

### 2.4. Questionnaires to Evaluate Student’s and Faculty’s Perceptions

Each e-lecture contained an embedded questionnaire that solicited students’ opinions about the important lecture elements: the quality/clarity of lecture information, audio narration, educational videos, animations, and embedded questions. This step was necessary to assist the instructors in identifying any technical problems/student dissatisfaction periodically and making immediate modifications.

At the end of the current course, all students were encouraged to participate in a voluntary survey to solicit their feedback about both PICkLE and iFEEL [35]. The survey was in the Arabic language and designed to evaluate student feedback anonymously. The survey was created using Google Forms^®^ and the survey link was sent by the instructor to the students. The survey consisted of three sections related to the PICkLE model, the iFEEL learning model, and general points/suggestions. In addition, data collected from the surveys distributed in the past live courses were analyzed and compared with the current course data.

Another anonymous, voluntary survey was sent to Pharmacy College faculty members at various Saudi Universities. The survey sample covered both governmental and private college faculty members and was intended to solicit their perceptions about iFEEL. The survey was created using Google Forms^®^ with the option of selecting an Arabic or English version, and the survey link was sent to the faculty university emails. The survey consisted of three sections: general respondent information, evaluation of an example of the designed e-lectures, and perceptions and opinions about using e-lectures as a model for flipped learning.

### 2.5. Validity and Reliability of the Surveys

Each questionnaire was reviewed linguistically and analytically to confirm that the presentation of questionnaire items was relevant to the study scope, clear, and unambiguous to understand and respond to [37]. The questionnaire Likert scale items were rated from 1 (I strongly object/not completely helpful, etc.) to 5 (I strongly support/very helpful, etc.) and subsequently assessed by descriptive analysis namely, the mean and standard deviations [38]. The homogeneity of the faculty’s responses across different general characteristics (gender, academic rank, degree, and discipline) was assessed by ANOVA and Levene’s tests [39,40]. The student’s and faculty’s written comments were subjected to qualitative content analysis to find common themes among the perceptions [41]. The reliability (internal consistency) of the surveys was assessed through Cronbach’s alpha (α) value. An α value above 0.7 was considered acceptable [36,37,42].

### 2.6. Ethical Considerations

The protocol for this research was reviewed and approved by the KSU Medical College Institutional Review Board (#E-21-5718). Retrospective analyses of student scores and feedback were collected as part of normal assessment during the course. Students were informed in advance that the e-lectures contained an embedded survey to assess the quality of each lecture and identify any technical issues. Student course completion and faculty members’ surveys included a pre-statement that the survey was voluntary and anonymous.

### 2.7. Statistical Analysis

The analysis was conducted using the Statistical Package for the Social Sciences (SPSS) version 26 (IBM, Armonk, NY, USA). The ANOVA followed by LSD and independent *t*-test were used to compare students’ scores and attendance. A *p*-value of ≤0.05 was considered statistically significant.

## 3. Results

A total of 158 students were involved in the current study. One hundred forty-nine studied the course live from 2018–2020 and nine students took the course remotely in the spring 2021 semester. In the current remote course offering, 88.9% of students were able to complete the e-lectures with more than 80% score on time. Furthermore, the average attendance was 97.8% for PICkLE and 95.6% for IFEEL activity (Table 1). Student mean scores significantly increased from 16.4% (pre-test) to 93.5% (post-test) on the PiCkLE activity and from 22.2% (pre-test) to 95.2% (post-test) on the iFEEL activity (*p* < 0.05). Interestingly, mean comprehensive exam scores increased from 83.8% (for PICkLE) to 89.2% (for iFEEL). Specifically, mean post-test scores were higher in the iFEEL activity (95.2%) compared to both remote PICkLE (93.5%, *p* = 0.658) and live PICkLE (90.2%, *p* = 0.08). When comparing attendance, student scores in the pre-test, post-test, and comprehensive exams, there was no significant difference between the two activities (Table 1).

A total of 93 students completed the surveys which represented an 85.3% response rate of the total students who were surveyed (n = 109). In general, both models showed high student acceptance. Compared to remote PICkLE, student responses were overwhelmingly more positive for the iFEEL activity as shown in Table 2 and Table 3.

The ANOVA and Levene’s tests showed no significant differences (*p* > 0.05) of faculties perceptions across different gender, academic ranks, degrees, and disciplines. These findings confirm the homogeneity of the responses across these general characteristics. The Cronbach’s alpha (α) values for different students and faculty survey sections ranged from 0.783 to 0.912 indicating high internal consistency and reliability of the utilized tools.

Regarding the quality of the interactive e-lectures, 90–95% of students agreed/strongly agreed, with the clarity, quality, and benefits obtained from the audio explanation, educational videos, animated illustrations, and embedded questions (Table 2).

Interestingly, 94.1%, 62.5%, and 87.5% of the participants recommended that future offerings of the course incorporate the live PICkLE, remote PICkLE, and iFEEL activities, respectively. Regarding the live PICkLE activity, 88% and 70% confirmed the usefulness of the live PICkLE activity and their ability to remember the basic information studied in this activity, respectively. Similarly, 100% and 87.5% of the students felt that the remote PICkLE and iFEEL activities helped them to understand the lecture material, respectively (Table 3). Furthermore, 87.5% reported that the iFEEL activity was useful/highly useful in motivating them to review the lecture materials before the class and motivating them to focus during the lecture (Table 3); while 87.5% responded that the remote PICkLE activity was useful/highly useful in improving their inter-personal skills.

Students were also asked to compare the two models according to their preferences. When asked which model they preferred in terms of understanding the information, 75% preferred the iFEEL activity, 12.5% preferred the PICkLE activity, and 12.5% felt that both activities were equally the same. Interestingly, the student preference further increased towards iFEEL when they were asked about their preferences in terms of visualizing the equipment and their methods of operation. In this point, 87.5% preferred the iFEEL and 12.5% felt they were both the same (Table 3).

Regarding the post-tests, which were conducted in both learning models, 100% and 75% of students reported that the post-tests were useful/very useful in helping them understand key information and encouraging them to stay focused during the lecture, respectively (Table 3).

In respect to the faculty survey, thirty-two faculty members completed the survey; 62.5% were males, 69% were assistant professors and 87.5% were PhD holders (Table 4). Although the response rate for the faculty survey was very low, the responses represented nearly 50% of the Saudi universities with pharmacy/health care colleges.

Similar to the student survey, the faculty survey also showed high acceptance of the interactive e-lectures model. Regarding the quality of the interactive e-lectures, 78–94% of faculty members agreed/strongly agreed with the clarity, quality, and benefits obtained from the audio explanation, educational videos, animated illustrations, and embedded questions (Table 2).

Regarding using e-lectures as a model for flipped learning, 90% and 87% of faculty members reported that this model is helpful/very helpful in motivating students to preview the lecture material before attending the lecture and focus during the lecture, respectively (Table 5). In addition, 94% of faculty members perceived the “educational videos and animated illustrations” to be useful/very useful in understanding the lecture material (especially the material that requires practical skills or visual illustrations) (Table 5).

Interestingly, only 14% of faculty members regularly used e-lectures before the COVID-19 pandemic, 57% used it during the pandemic, and 29% never used it. However, 84% supported/strongly supported the integration of this type of interactive e-lectures in the courses they teach (Table 5). To explore students’ and faculties’ perceptions, a qualitative content analysis was employed to analyze the open-ended responses. After revision and refinement, the process yielded the most important themes captured from students’ and faculties’ responses. The results are described in Table 6, Table 7 and Table 8.

## 4. Discussion

In the current study, the PICkLE and iFEEL activities were evaluated for their potential enhancement of student engagement prior to and within the lecture. iFEEL embodied the qualities of a high-impact e-learning activity during the COVID-19 pandemic [22]. Students and faculty perceived that the content was presented in an effective and user-friendly format that engaged the students. Student learning was supported by synchronous in-class activities, and the PICkLE activity could always be used as a contingency plan if technical difficulties arise.

In live lectures, it is easy to facilitate group learning activities with no significant interruption from other group members. However, in online learning, implementation of the PICkLE activity faced some difficulties because each group of students had to enter a separate, unsupervised virtual meeting room to avoid interruption from their colleagues in the other groups. After completing the in-class group test, students had to come back again to the instructor’s main virtual room to complete the discussion. This challenge, along with occasional intermittent internet connection problems, hindered the smooth flow of the synchronous online discussions. Wong and Kan reported that maintaining effective interaction, among students and with the instructors, in small online group learning could be challenging [9]. Duszenko et al. reported that some students faced technical difficulties and poor interaction between group members during online group work. Accordingly, 54% of students opposed the notion that online teamwork helped them understand the topic [11]. Furthermore, Kalmar et al. found that students reported lowered productivity in group work and difficulty in receiving feedback from peers after switching to online education [43]. This could explain the reason for the relatively lower student preference using remote PICkLE activity versus the live PICkLE in future course offerings (Table 3). On the other hand, the iFEEL activity involved interactive embedded questions in the e-lecture which strengthened the students’ comprehension of the lecture material and helped them to take the in-class lecture test, individually. This can be confirmed by the instructor’s observation that the students required less time for taking the in-class test and/or discussing their results. However, both techniques had a positive influence on student attendance, participation, and exam scores. These encouraging results led to the incorporation of the developed e-lectures in the subsequent offering of the Pharmaceutical Quality Control and GMP course.

The high attendance rate, in both activities, indirectly reflects the acceptance of students of both methods in the current emergent distance-learning situation. Moreover, the high rate of student participation in the iFEEL activity reflects the high student acceptance and the lack of serious technical problems faced by students while reviewing the e-lectures. Rodrigues et al. also noted that e-learning was associated with higher student attendance in a systematic review conducted prior to COVID-19 [44]; however, Abbasi et al. reported that healthcare students expressed difficulties with course schedules during the pandemic [45]. These obstacles may have been more profound among distance learning naïve students than those who were more technologically literate [46,47].

An improvement in students’ marks is generally an indication that the students achieved the course-related learning outcomes. In this respect, the post-tests student scores increased by 5.7- and 4.2-folds (*p* < 0.05) compared to pre-tests in PICkLE and iFEEL, respectively. These findings are in agreement with a recent study that evaluated an e-learning course with computer-assisted simulation materials. The study showed a significant increase in the mean post-test score compared to that of the pre-test [36]. These findings imply that both iFeel and PICkLE were successful in improving students’ comprehension and learning outcomes.

The post-tests were very useful in helping students to understand the most important information and motivating them to focus during the lecture (Table 3). Similarly, several previous studies showed positive educational outcomes of “End of class” (post-lecture) tests in significantly increasing average exam scores [48], encouraging student introspection related to their personal comprehension [49,50], as well as improvements in student course satisfaction [51].

In our study, the iFEEL showed a non-significant increase in mean comprehensive exam score and post-test scores compared to PICKLE. These results are parallel to the findings of Wilson et al. that showed a non-significant increase in student performance when comparing a flipped classroom activity with traditional teaching methods [32]. The high percentage and similarities in student attendance and scores are indications that both PICkLE and iFEEL activities are non-inferior to each other and can be an alternative to the standard didactic lectures as a part of either live or distant learning courses. A recent study also concluded that a non-significant increase in student exam scores when comparing two different active learning activities is a positive indication that both strategies are equally effective in enhancing student learning and understanding as an alternative to traditional lectures [50]. Moreover, mean post-test scores were non-significantly higher in the iFEEL activity compared to pre-pandemic live PICkLE. Although no significant difference was found, these findings confirm that the adopted e-course was at least as effective for content knowledge as our pre-COVID face-to-face course. Duszenko et al. reported that none of the e-learning instructed students performed worse than their face-to-face instructed colleagues [11].

A recent study that implemented similar interactive e-lectures reported some technical issues such as tone quality, difficulties playing lectures (including using the full-screen mode on some equipment and incompatibility with PC operating system), or technical abilities of students (e.g., installing relevant software) [11]. This highlights the importance of evaluating the quality attributes of e-lectures periodically. In our study, the majority of students agreed/strongly agreed that the audio, videos, animated illustrations, and embedded questions were clear and of good quality. No student had negative comments about the quality of any element of the lecture and none reported any technical concerns.

Student perceptions of the iFEEL activity were more favorable than the PICkLE activity. Most students (78.5%) preferred the iFEEL over the PICkLE for future course offerings, particularly in terms of visualizing the equipment and their operation. This data represents a significant improvement in students’ acceptance of flipped learning compared to previous studies [52,53]. Wilson et al. found that only 5.7% of students preferred the flipped method alone [32]. Cho and Kim reported that students showed inferior self-directed learning readiness and instructor–student interaction in online flipped learning compared to their counterparts who received face-to-face flipped learning [10].

Most students affirmed the benefit of PICkLE for understanding the lecture and developing their personal skills. Similarly, Bouw et al. reported that students had positive perceptions toward student-led team-based learning activities and considered them as an effective method of peer-to-peer teaching [54]. Valler-Jones concluded that students stated that peer-led activities enhance many essential skills such as communication, teamwork, and leadership [55].

Similar to the students’ feedback, most faculty members considered the iFEEL activity to be of good quality and believed that it had a high potential to encourage student engagement with the lecture material both before and within the class. The increased percentage of faculty members that utilized interactive e-lectures during the COVID-19 pandemic may indicate the possible future acceptance of such a model in their regular teaching practices.

During the COVID-19 pandemic and the emergent distant learning, virtual cloud lectures were plagued by some technical issues such as the intermittent internet connection faced by some students [45,56,57]. Alternatively, some faculty chose to send their recorded videos or PowerPoint slides to the students [1,58]. A common drawback of these methods is that they are non-interactive, primarily instructor-focused, and lack the instructor’s supervision and/or control of how much time students spend reviewing the lecture materials. Even with the Blackboard feedback feature to monitor student access to the posted content, some students might just play the video and not pay attention to lecture explanation. These frustrations were echoed in many published studies conducted during the pandemic. Healthcare students preferred traditional lectures due to the lack of application of clinical skills, minimal opportunities for interaction, the poor delivery methods of technologically-naïve faculty members, vague or inadequate policies for online classes, exams, and grade distribution, limitations on exam times, and difficulty concentrating [45,46,57,59,60,61,62,63,64,65]. Recommendations for improvement included combining traditional teaching methods and blended learning, more interaction during lectures, and better-designed e-courses [45,62,66]. In our study, an attempt was made to overcome these obstacles with the utilization of the iFEEL activity. The interactive e-lectures were restricted to prevent the student from skipping any slide if he did not spend the pre-determined time on the slide, and/or interact with the required slide items. In addition, several embedded questions were distributed throughout the e-lecture slides. Student participation was only considered if he received a minimum of 80% score by the specified deadline. These factors together could have enhanced student focus during lecture review, step-by-step comprehension, and his interaction with the lecture materials. During the lecture period, students had to complete an individual open-book exam followed by a debriefing session with the instructor. iFEEL has several additional advantages including the use of technology to help students visualize the lecture material prior to the lecture and the ability to partially overcome the internet limitations through decreasing the time spent in live streaming of the lecture explanation and focusing more on discussion and activities. Moreover, this method also allows the students to study the lecture material at a suitable time based on their daily schedule. Pilkington and Hanif reported that the asynchronous sessions were more equitable than synchronous ones because students with difficult and challenging home/learning environments (such as disruptions at home, limited access to devices, poor internet, etc.) were minimally disadvantaged [1]. Duszenko et al. reported that students valued the greater flexibility and time efficiency, student-centered format (particularly for flipped classrooms) offered by an online learning environment [11]. These findings indicate that iFEEL has great potential for enhancing student learning outcomes and promoting an increased level of faculty involvement. Förster et al. reported many advantages of e-learning mentioned by lecturers, and, interestingly, all were in agreement with the views of the post-graduate medical trainees. The advantages included the flexibility and compatibility with each individual’s lifestyle as well as cost-savings and the elimination of hardships associated with commuting [63]. However, it should be noted that previous research also suggested that a supportive learning environment played a key role in the successful student adaptation of online education [4].

Several limitations should be discussed. Due to the limited number of current course participants, the results of the study may not be generalizable. Future studies should include larger samples, multiple class cohorts, or students from multiple universities. In this study, the designed e-lectures consumed a significant amount of effort and time to prepare. This might hinder faculty acceptance to apply such interactive e-lectures in their courses. Future steps should involve integration with information technology unit in order to facilitate the fast design of the e-lectures.

We recommend that future studies are designed to assess the long-term effects of iFEEL on knowledge retention and to define course types that are best suited for this activity. Furthermore, the study of benefits and burdens upon the faculty is necessary in order to facilitate implementing such e-learning activities. This study provides another active learning resource that can be incorporated into distance as well as blended learning, during and after the COVID-19 pandemic.

## 5. Conclusions

Educators must continue to strive to ensure that student comprehension and retention remain paramount. iFEEL represents a novel model of remote flipped learning and shows promising potential to be incorporated into online learning activities. The current model can be applied in healthcare courses and other disciplines that require a visual explanation and/or practical skills. Both faculty and students held positive opinions about the iFEEL activity. Furthermore, iFEEL post-test student scores significantly improved when compared to their pre-test results, and students preferred this activity over the PICkLE activity. In addition, the significant improvement in student pre-test scores and the non-significant differences between the iFEEL and PICkLE activities are indications that both activities can be potentially incorporated into distance learning courses. Developing distance-based engaging learning techniques, such as iFEEL, is essential for the current and future learning environments.

## Figures and Tables

**Table 1:** Students’ average attendance, participation, and exam score.

Teaching Technique	Average Attendance (%) *	Mean Pre-Test Score (%)	Mean Post-Test Score (%)	Mean Comprehensive Exam Score (%)
Live Paper-based learning method (PICkLE), n = 149	NA	NA	90.2 ± 8.26	NA
Remote Paper-based learning method (PICKLE), n = 9	97.8 ± 5.0	16.4 ± 17.1	93.5 ± 8.4	83.8 ± 18.7
Remote Interactive-electronic learning method (iFEEL), n = 9	95.6 ± 9.9	22.2 ± 16.3	95.2 ± 7.7	89.2 ± 9.2
Statistical test	Independent *t*-test	Independent *t*-test	ANOVA followed by LSD	Independent t-test
*p*-value	0.667	0.474	iFEEl vs. live PICkLE, (0.08), iFEEl vs. remote PICkLE (0.658)	0.449

* Data are presented as percentage of total class students (mean ± SD).

**Table 2:** Students’ and faculty members’ perceptions on the five quality attributes of the interactive e-lecture.

Question **	Students (Responses = 40), Faculty (Responses = 32)	I Strongly Support (%) *	I Support (%) *	Neutral (%) *	I object (%) *	I Strongly Object (%) *	Mean	Standard Deviation
The lecture material was clear and easy to understand	Students	72.5%	22.5%	5%	0	0	4.68	0.57
	Faculty	31.3%	56.3%	12.5%	0	0	4.19	0.64
The audio quality and illustrations were clear	Students	65.0%	27.5%	7.5%	0	0	4.58	0.64
	Faculty	37.5%	56.3%	6.3%	0	0	4.31	0.59
The lecture animation helped me to understand the lecture material	Students	65.0%	25.0%	10.0%	0	0	4.55	0.68
	Faculty	25.0%	53.1%	18.8%	0	0	4.00	0.76
The video quality was good	Students	72.5%	22.5%	5%	0	0	4.68	0.57
	Faculty	34.4%	50.0%	12.5%	0	0	4.16	0.77
The embedded questions were interactive and helped me to understand the lecture	Students	72.5%	17.5%	10%	0	0	4.63	0.67
	Faculty	31.3%	50.0%	15.6%	0	0	4.09	0.78

* Data are presented as the percentage of total class students’ and faculty responses. ** Questions were rephrased to combine both students and faculty members’ responses.

**Table 3:** Students’ perceptions about the live PICkLE, remote PICkLE, and iFEEL activity.

**Question**	**Learning Method**	**I Strongly Support** (%) *	**I Support** (%) *	**Neutral** (%) *	**I Object** (%) *	**I Strongly Object** (%) *	**Mean**	**Standard Deviation**
Do you support incorporating these learning models in future course offerings?	Live PICkLE (n = 84)	77.4%	16.7%	4.8%	1.2%	0	4.70	0.62
	Remote PICkLE (n = 8)	62.5%	0	12.5%	25%	0	4.00	1.41
	iFEEL (n = 8)	75%	12.5%	0	12.5%	0	4.50	1.07
**Question**	**Learning Method**	**Excellent** (%) *	**Good** (%) *	**Acceptable** (%) *	**Weak** (%) *	**Very Weak** (%) *	**Mean**	**Standard Deviation**
How well do you remember the basic information you studied in the course?	Live PICkLE (n = 85)	12.9%	57.6%	24.7%	2.4%	2.4%	3.76	0.80
How would you rate your understanding of the lecture material?	Remote PICkLE (n = 8)	62.5%	37.5%	0	0	0	4.63	0.52
	iFEEL (n = 8)	75%	12.5%	12.5%	0	0	4.63	0.74
**Question**	**Learning Method**	**Very** **Useful** (%) *	**Useful** (%) *	**Neutral** (%) *	**Not Useful** (%) *	**Absolutely Useless** (%) *	**Mean**	**Standard Deviation**
How useful was studying this course in groups?	Live PICkLE (n = 85)	57.6%	30.6%	8.2%	1.2%	2.4%	4.40	0.88
How useful was this model in developing your personal skills (teamwork - the ability to negotiate and persuade–decision-making)?	Remote PICkLE (n = 8)	50%	37.5%	12.5%	0	0	4.38	0.74
How useful was the “e-lectures” in motivating you to review the scientific material before attending the lecture?	iFEEL (n = 8)	75%	12.5%	12.5%	0	0	4.63	0.74
How useful was the “e-lectures” in motivating you to focus during the lecture?	iFEEL (n = 8)	75%	12.5%	12.5%	0	0	4.63	0.74
**Question**			Remote PICkLE *	iFEEL *		Both are Same *	None of Them *	
Which method do you prefer to use in teaching this course in terms of clarification and retention the information? (n = 8)			12.5%	75%		12.5%	0	
Which method do you prefer to use in teaching this course in terms of visualizing the instruments and how they work? (n = 8)			0	87.5%		12.5%	0	

* Data are presented as the percentage of total class students’ responses (n = 8).

**Table 4:** Faculty demographics.

**Gender**	Male *		Female *		
	62.5%		37.5%		
**Academic rank**	Professor *	Associate Professor *	Assistant Professor *	Lecturer *	Teaching Assistant *
	3.1%	15.6%	68.8%	12.5%	0
**Academic Degree**	Ph.D. *	M.Sc. *	Pharm. D *	Bachelor *	M.D *
	87.5%	12.5%	0	0	0

* Data are presented as the percentage of total faculties’ responses (n = 32).

**Table 5:** Evaluation of using e-lectures as a model for flipped learning by faculty.

**Question**	**Very Helpful** (%) *	**Helpful** (%) *	**Neutral** (%) *	**Not Helpful** (%) *	**Not** Completely Helpful (%) *	**Mean**	**Standard Deviation**
What is the expected effect of using this model in motivating the student to peruse the scientific material before attending the lecture? (n = 32)	25.0	65.1	3.1	6.3	0	4.09	0.73
What is the expected effect of using this model in motivating the student to focus during the lecture? (n = 32)	40.6	46.9	9.4	3.1	0	4.25	0.76
**Question**	Very Useful (%) *	Useful (%) *	Neutral (%) *	Not Useful (%) *	Not Completely Useful (%) *	Mean	Standard Deviation
How useful do you consider the “visual instructional videos and animated illustrations” in understanding the material (especially the one that needs practical skill or visual illustration)? (n = 32)	53.1	40.6	6.3	0	0	4.47	0.62
**Question**	I Strongly Support (%) *	I Support (%) *	Neutral (%) *	I Object (%) *	**I Strongly** Object (%) *	Mean	Standard Deviation
Do you support the integration of this type of interactive e-lectures in the courses you teach? (n = 32)	37.5	46.9	9.4	3.1	3.1	4.13	0.94

* Data are presented as the percentage of total faculties’ responses.

**Table 6:** Advantages and Disadvantages of paper-based in class group learning (PICkLE) from students’ point of view.

Item	Initial Codes	Number of References	Main Themes
**Advantages**	Learning by extracting information and answering questions Learning ways to search for information	2	Self-directed learning
	Deep understanding Ease access to the information Simplified in a way that does not exclude crucial information	2	Effective comprehension
	Teamwork Decision-making Responsibility Peer learning	2	Enhancing interpersonal skills
**Disadvantages**	Time consuming Inappropriate class schedule	2	Time-related issues
	Language difficulty A lot of reading	1	Difficulty in comprehension
	Not (physically) dealing with devices considering that we have dealt with them in previous courses	1	Lack of hands-on training for practical aspects

**Table 7:** Advantages and disadvantages of interactive flipped e-learning activity (iFEEL) from students’ points of view.

Item	Initial Codes	Number of References	Main Themes
**Advantages**	Concise, useful, and clear Not boring Explanation with examples, questions and testing quickly	3	Appropriate format/structure
	Time flexibility Ability to re-access the lecture anytime	1	Flexible ways of learning
**Disadvantages**	Not (physically) dealing with devices considering that we have dealt with them in previous courses	1	Lack of hands-on training for practical aspects
	Not taking it seriously	1	Lack of dedication

**Table 8:** Advantages and disadvantages of interactive flipped e-learning activity (iFEEL) from faculty points of view.

Item	Initial Codes	Number of References	Main Themes
**Advantages**	Current generation suitability Providing ways for different learning styles	2	Modern Teaching/learning strategy
	Clearance and simplicity Information flow Higher level of comprehension Visualization Good for the practical aspect of the course	12	Appropriate format/structure
	Draws the student’s attention to the lecture information Motivates students to interact with the lecture questions Increases fun during learning Motivates students to prepare for the lecture, focus during the lecture, and interact with the instructor and his colleagues Allows more time for activities and exercises during the class Engaging	9	Engaging and Interactive
	Student can study/revise the lecture at his convenience	3	Flexibility
	Student-centered and self-directed learning	3	Student-centered
	Better time management for the students and the instructors	2	Time effective
**Disadvantages**	Preparation and execution are time consuming for the instructors Time consuming for the student	5	Time-related issues
	Software costs Internet issues Requires technical skills	4	Technical issues
	Not applicable to all topics particularly theory extensive courses The design is suitable for self-education and work-shops rather than academic activities	4	Subject/topic issues
	Monitoring system is required to check compliance	4	Compliance issues
	Not taking into account individual differences among students Limits direct contact and live interaction with the instructor Completing the e-lecture does not mean that the student benefited from the lecture material	5	Poor comprehension and interaction

## Data Availability

The data that support the findings of this study are available from the corresponding author, [AAS], upon reasonable request.
